# Identifying Interactions between Dietary Sodium, Potassium, Sodium–Potassium Ratios, and *FGF5* rs16998073 Variants and Their Associated Risk for Hypertension in Korean Adults

**DOI:** 10.3390/nu12072121

**Published:** 2020-07-17

**Authors:** Hyeyun Jeong, Hyun-Seok Jin, Sung-Soo Kim, Dayeon Shin

**Affiliations:** 1 Department of Food and Nutrition, Inha University, 100 Inha-ro, Michuhol-gu, Incheon 22212, Korea; livelys@naver.com; 2 Department of Biomedical Laboratory Science, College of Life and Health Sciences, Hoseo University, Asan, Chungnam 31499, Korea; jinhs@hoseo.edu (H.-S.J.); kims7152@naver.com (S.-S.K.)

**Keywords:** Korean Genome and Epidemiologic Study (KoGES), genetic variants, sodium, potassium, sodium-potassium ratios, hypertension

## Abstract

Hypertension is affected by both genetic and dietary factors. This study aimed to examine the interaction between dietary sodium/potassium intake, sodium–potassium ratios, and *FGF5* rs16998073 and link these with increased risk for developing hypertension. Using data from the Health Examinee (HEXA) Study of the Korean Genome and Epidemiologic Study (KoGES), we were able to identify a total of 17,736 middle-aged Korean adults who could be included in our genome-wide association study (GWAS) to confirm any associations between hypertension and the *FGF5* rs16998073 variant. GWAS analysis revealed that the *FGF5* rs16698073 variant demonstrated the strongest association with hypertension in this population. Multivariable logistic regression was used to examine the relationship between dietary intake of sodium, potassium, and sodium–potassium ratios and the *FGF5* rs16998073 genotypes (AA, AT, TT) and any increased risk of hypertension. Carriers with at least one minor T allele for *FGF5* rs16998073 were shown to be at significantly higher risk for developing hypertension. Male TT carriers with a daily sodium intake ≥2000 mg also demonstrated an increased risk for developing hypertension compared to the male AA carriers with daily sodium intake <2000 mg (adjusted odds ratio (AOR) = 2.41, 95% confidence intervals (CIs) = 1.84–3.15, *p*-interaction < 0.0001). Female AA carriers with a daily potassium intake ≥3500 mg showed a reduced risk for hypertension when compared to female AA carriers with a daily potassium intake <3500 mg (AOR = 0.75. 95% CIs = 0.58–0.95, *p*-interaction < 0.0001). Male TT carriers in the mid-tertile for sodium–potassium ratio values showed the highest odds ratio for hypertension when compared to male AA carriers in the lowest-tertile for sodium–potassium ratio values (AOR = 3.03, 95% CIs = 2.14–4.29, *p*-interaction < 0.0001). This study confirmed that *FGF5* rs16998073 variants do place their carriers (men and women) at increased risk for developing hypertension. In addition, we showed that high daily intake of sodium exerted a synergistic effect for hypertension when combined with *FGF5* rs16998073 variants in both genders and that dietary sodium, potassium, and sodium–potassium ratios all interact with *FGF5* rs16998073 and alter the risk of developing hypertension in carriers of either gender among Koreans.

## 1. Introduction

Hypertension is one of the most prevalent chronic diseases in the world [1] and is well known to cause various cardiovascular diseases including arterial stiffness, stroke, and heart disease [2]. The prevalence of hypertension in those over 30 years of age is 32.4% for men and 21.3% for women in Korea [3] and the annual cost of treating hypertension is approximately 3332.9 billion won [4]. Thus, the treatment and prevention of hypertension is one of the top priorities for public health in Korea and the rest of the world.

Hypertension is affected by both genetic and environmental factors including diet, especially sodium and potassium intake. Studies have revealed a positive interaction between sodium intake and increased blood pressure [5,6]. Increased potassium consumption has been shown to increase urinary sodium excretion [7] and decrease the risk of developing hypertension [5]. A meta-analysis showed that the sodium–potassium ratio is a more reliable index of the risk of hypertension than either sodium or potassium alone [8]. However, the use of the sodium–potassium ratio as a risk index for hypertension is still controversial, although there are documented beneficial effects of low sodium-to-potassium ratios, reduced sodium intake, and increased potassium intake including a study on the Dietary Approaches to Stop Hypertension (DASH) diet, which showed a reduction in blood pressure in both hypertensive and normotensive populations [9].

Fibroblast growth factor (*FGF*) signaling plays an important role in endothelial homeostasis [10] even though there have not been many studies demonstrating direct interactions between blood pressure and *FGF5*. However, several previous genome-wide association studies (GWAS) have shown that the single nucleotide polymorphism (SNP) rs16998073 found in the *FGF5* gene, a member of the *FGF* family, is strongly associated with hypertension in East Asians and Europeans [11,12,13].

Associations between SNPs (*AGT* rs699, *ADD1* rs4961, *NPPA* rs5063, *GPX1* rs1050450, *AGTR1* rs5186, *CSK* rs1378942, *CSK-MIR4513* rs3784789, *LOC101929750* rs7554672, *MKLN1* rs1643270, *TENM4* rs10466739, *ATP2B1* rs17249754) and sodium/potassium intake and the sodium–potassium ratio and their effects on the risk of hypertension have been studied previously [14,15,16]. However, no studies have evaluated the interaction between *FGF5* rs16998073 and dietary factors stratified on their recommended daily intake values. Therefore, our objective was to determine the risk of hypertension associated with dietary factors and *FGF5* rs16998073 by evaluating the interactions between this SNP, the intake of sodium/potassium, the sodium–potassium ratio, and the occurrence of hypertension in middle-aged Koreans of both genders.

## 2. Materials and Methods

### 2.1. Data Source and Study Population

Data were obtained from the Health Examinee (HEXA) Study, a large-scale community-based prospective ongoing cohort study that forms part of the Korean Genome and Epidemiologic Study (KoGES). The HEXA study included 173,357 participants, all of whom were older than 40. Using the baseline data from the HEXA cohort, we went on to perform a series of novel cross-sectional analyses. This study was conducted between 2004 and 2013 by the Korean Centers for Disease and Control (KCDC) to explore dietary and lifestyle factors and their effect on chronic diseases in the Korean population.

**Figure 1:**
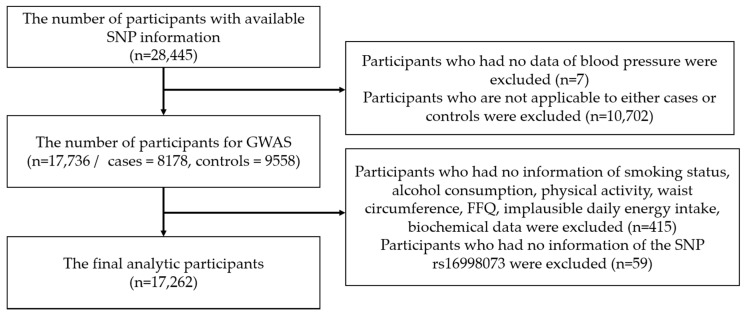
Process flow chart outlining the relevant steps for this analysis. SNP: single-nucleotide polymorphism, GWAS: genome-wide association study, FFQ: food frequency questionnaire.

Figure 1 is a flow chart describing the selection process for this study. Detailed descriptions of the selection process used in this study can be found below.

We used GWAS to test the association between all the SNPs and an increased risk for hypertension. From a total of 28,445 participants with available SNP information, participants who had missing blood pressure data (*n* = 7) and those who did not belong to the condition of the cases or controls (*n* = 10,702) were excluded due to no diagnosis of hypertension or blood pressure condition. This left a total of 17,736 participants (cases = 8178, controls = 9558) eligible for GWAS analysis.

After completing the GWAS analysis, any participants without information on smoking status (*n* = 73), alcohol consumption (*n* = 37), physical activity (*n* = 19), waist circumference (*n* = 7), food frequency questionnaire (FFQ) (*n* = 195), implausible daily energy intake (<500 and ≥4000 kcal) (*n* = 45), or biochemical data (*n* = 39) were all excluded. Participants without an *FGF5* rs16998073 genotype (*n* = 59) were also excluded. This left 17,262 participants eligible for downstream analyses. These participants were evaluated for interactions between genetic and dietary factors and their effect on the risk of hypertension. The study protocol was reviewed and approved by the Institutional Review Board (IRB) at Inha University (IRB approval no.: 200129-1A). This study was conducted using biosources from the National Biobank of Korea at the Centers for Disease Control and Prevention in the Republic of Korea (KBN-2020-016).

### 2.2. General Characteristics, Anthropometric, and Biochemical Variables 

Information on age, smoking habits, alcohol consumption, and physical activity were collected using a structured questionnaire by trained interviewers. Height was measured to the nearest 0.1 cm while the patients were barefoot or wearing thin socks, and the subject was placed upright on a horizontal surface. Weight was measured to the nearest 0.01 kg wearing light clothing. Body mass index (BMI) was calculated by dividing weight (kg) by the square of the height (m^2^). Waist circumference was measured using a tape measure circumventing the body between the lowest ribs and the iliac ridge, the tape measure was held loosely enough to allow minimal contact when the patient exhaled normally. Blood samples were collected in the morning after at least 8 h of fasting. Serum total cholesterol, triglyceride, and high-density lipoprotein (HDL) cholesterol levels were measured using an automatic analyzer (ADVIA 1650, Siemens, Tarrytown, NY, USA).

### 2.3. Dietary Assessment 

Dietary information was collected using a food frequency questionnaire (FFQ), which evaluates the frequency and intake of 106 common foods consumed by Koreans over the past year from the time of investigation. The FFQ is composed of nine ranges for intake frequency with each divided by three serving sizes. It is possible to calculate the average daily intake of various nutrients using the information supplied by the FFQ. The daily intake of each food item was calculated using the frequency of consumption and the serving size for each food, and this was then used to calculate daily sodium and potassium intake per person per day. Calculation of nutrient intake was based on the Food Composition Table in the Recommended Dietary Allowances for Koreans [17]. The sodium–potassium ratio was calculated by dividing the sodium intake by the potassium intake. Further information on the validity and reproducibility of the FFQ can be found elsewhere [18,19].

### 2.4. Ascertainment of Hypertension

Blood pressure measurements were taken in a stable, comfortable, warm (approximately 25 °C) seated position, with both arms held at the height of the heart. Each blood pressure measurement was taken at least twice with at least 5 min between the readings. If these blood pressure readings differed by more than 5 mmHg, the blood pressure was reevaluated until two similar measurements were obtained. Participants who had a systolic blood pressure (SBP) ≥140 mmHg or a diastolic blood pressure (DBP) ≥90 mmHg and any patient receiving any antihypertensive medication were defined as hypertensive whether they had a clinical diagnosis of hypertension or not. Normotensive controls were those individuals with no medical history of hypertension and with an SBP <120 mmHg and a DBP < 80 mmHg.

### 2.5. Genome-Wide Genotyping and Quality Control

The detailed information for this protocol has been described elsewhere [20]. Genomic DNA samples were genotyped using the Axiom^®^ 2.0 Reagent Kit (Affymetrix Axiom^®^ 2.0 Assay User Guide) according to the manufacturer’s protocol. The genotype data were produced using the K-CHIP, which was available through the K-CHIP consortium. Samples with the following features were excluded during the quality control process: sex inconsistency (the difference between the assigned sex and the sex determined based on the genotype [21]), markers with a high missing rate (>5%), individuals with a high missing rate (>10%), minor allele frequency <0.01, and a significant deviation from Hardy–Weinberg equilibrium (HWE) (*p* < 0.001). In addition, SNPs were excluded if they were related to each other in linkage disequilibrium.

After genotyping and sample quality control, GWAS was performed to select SNPs associated with hypertension using PLINK version 1.9 beta [22]. To investigate the interaction between each SNP and the risk of hypertension, we conducted a multivariable logistic regression after controlling for age and gender. We also performed a linear regression to determine whether there was any association between specific SNPs and blood pressure values. This evaluation yielded 23 SNPs demonstrating genome-wide significance (*p* value < 5 × 10^−8^), including *FGF5* rs16998073, which were included in this study.

### 2.6. Statistical Analyses

Categorical variables were evaluated using the Pearson chi-square test, whereas continuous variables were examined using the Mann–Whitney U test. The mean values and their standard deviations for all the continuous variables and the prevalence of each of the categorical variables were calculated.

The subjects were split into two groups on the basis of their daily intake of dietary sodium and potassium. The criteria for each group was based on the Dietary Reference Intake for Koreans 2015 [23]; which recommends a daily intake of 2000 mg for sodium and 3500 mg for potassium. The sodium–potassium ratio was categorized into tertiles. Multivariable logistic regression was performed to examine the interaction between *FGF5* rs16998073 and dietary factors with the risk of hypertension after controlling for covariates. Covariates included sex, age, examination site, smoking status, alcohol consumption, physical activity, and BMI to minimize the effect of confounding factors. All statistical analyses were performed using SPSS (version 25; SPSS Inc., IBM, New York, NY, USA) and a *p* value of <0.05 was considered significant.

## 3. Results

GWAS analysis revealed a total of 23 SNPs variants to exhibit some degree of genome-wide significance, suggesting that all these SNPs were significantly associated with hypertension. Table 1 shows the results of the GWAS; rs116998073, rs145808, rs36034102, rs112735431 were associated with a significantly increased risk of developing hypertension. The other 19 SNPs exhibited a protective effect on the risk of hypertension. Of these *FGF5* rs16998073 was shown to exhibit the strongest interaction between sodium and potassium intake and sodium–potassium ratios, genotype, and hypertension. The descriptions of SNPs including each minor alleles, minor allele frequency in cases and controls, gene, adjusted odds ratio (AOR), systolic blood pressure, and diastolic blood pressure are presented in Table 1.

Table 2 describes the distribution of the demographic characteristics for each of the study subjects stratified by gender. In both men and women, hypertension was significantly more common in patients with increased age, BMI, waist circumference, and triglyceride levels. HDL-cholesterol levels were lower in both men and women with hypertension than in those without hypertension. Smoking status, physical activity, and alcohol consumption significantly differed based on hypertension status in the male participants. In women, smoking status and alcohol consumption significantly differed between hypertensive and non-hypertensive participants. Lower potassium intake and higher sodium–potassium ratios were also more common in women with hypertension.

Finally, we performed a logistic regression analysis to confirm the interaction between *FGF5* rs16998073 genotypes, sodium and potassium intake, and the risk of hypertension (Table 3). The strongest risk effect was observed in male carriers with the homozygous rs16998073 mutant TT allele with a daily sodium intake ≥2000 mg (AOR = 2.41, 95% confidence intervals (CIs) = 1.84–3.15, *p*-interaction < 0.0001) when compared to those with homozygous wild-type alleles and a daily sodium consumption <2000 mg. Compared to male carriers with the heterozygous rs16998073 AT allele with a daily sodium intake ≥2000 mg, the homozygote TT allele with a daily sodium intake <2000 mg showed greater risk for hypertension (AOR = 1.62, 95% confidence intervals (CIs) = 1.35–1.95, *p*-interaction < 0.0001 vs. AOR = 2.04, 95% confidence intervals (CIs) = 1.48–2.83, *p*-interaction < 0.0001). It is speculated that the effect of genetics has a greater influence on hypertension than that of diet. The results show that the risk of hypertension increases in subjects with the T allele at rs16998073 and a sodium intake ≥2000 mg per day in both men and heterozygote women. Men with a daily potassium intake <3500 mg who were homozygous for the TT allele of rs16998073 showed an increasing trend for hypertension when compared to participants carrying the wild-type AA and AT allele, respectively (AOR = 2.15, 95% CIs = 1.74–2.64, *p*-interaction < 0.0001; AOR = 1.48, 95% CIs = 1.30–1.68, *p*-interaction < 0.0001, respectively). It was also found that genetics prevail over the nutritional aspect when comparing the men with a daily potassium intake <3500 mg who were homozygous for the TT allele of rs16998073 to heterozygotes with a potassium intake ≥3500 mg (AOR = 1.48, 95% CIs = 1.09–2.01, p-interaction < 0.0001). Women with the minor T allele for rs16998073 had an increased risk for hypertension. Heterozygote carriers of the AT allele with a daily potassium intake ≥3500 mg exhibited a higher risk for hypertension than those with a potassium intake <3500 mg per day (AOR = 1.32, 95% CIs = 1.05–1.65, *p*-interaction < 0.0001 vs. AOR = 1.27, 95% CIs = 1.15–1.41, *p*-interaction < 0.0001). In contrast, female homozygote AA allele carriers with a daily potassium intake ≥3500 mg exhibited a reduced risk for hypertension (AOR = 0.75, 95% CIs = 0.58–0.95, *p*-interaction < 0.0001).

The interaction between sodium–potassium ratios, the *FGF5* rs16998073 variant, and the risk of hypertension is presented in Table 4. Men with the rs16998073 homozygote TT allele in the mid-tertile for their sodium–potassium ratio had about a threefold increased risk of hypertension compared to carriers of the AA allele in the lowest tertile of sodium–potassium ratios (AOR = 3.03, 95% CIs = 2.14–4.29, *p*-interaction < 0.0001). Men in the highest tertile of sodium–potassium ratios carrying the rs16998073 wild-type AA allele had significantly increased risk for hypertension when compared to those with the AA allele in the lowest tertile for sodium–potassium ratios (AOR = 1.34, 95% CIs = 1.45–2.24, *p*-interaction < 0.0001). In both men and women, carriers of the rs16998073 minor allele AT or TT were at significantly higher risk for hypertension (all *p* values < 0.05). However, the risk for hypertension does not always follow with the increase of the ratio in both genders. It seems that the tendency of prevalence might have affected the risk for hypertension.

**Figure 2:**
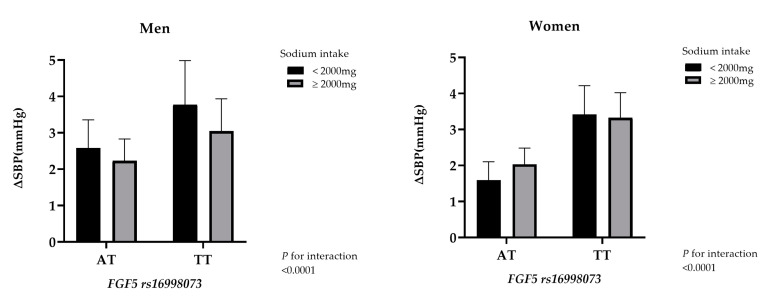
The bar graphs show the difference in SBP values in carriers with AT or TT variants separated by sodium consumption (<2000 or ≥2000 mg) and gender. The SBP value for the AA group was used as the reference. Error bars represent standard error. The *p* value for interaction for both genders was <0.0001.

The difference between the SBP values of homozygote wild-type (AA allele) and the other genotypes (AT or TT allele) stratified on sodium consumption is shown in Figure 2. Both men and women with the TT allele showed a greater difference in SBP values than their counterparts with the AT allele regardless of sodium consumption.

**Figure 3:**
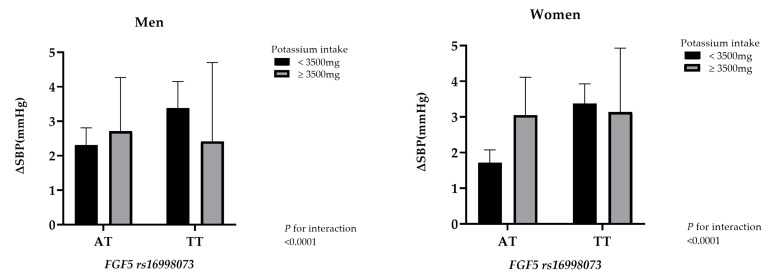
The bar graphs show the difference in SBP values in carriers with AT or TT variants separated based on potassium consumption <3500 and ≥3500 mg and gender. The SBP value for the AA group was used as the reference. Error bars represent standard error. The *p* value for interaction for both genders was <0.0001.

Figure 3 presents the difference in SBP values between homozygote wild-type (AA allele) and the other genotypes (AT or TT allele) separated according to potassium consumption. Both men and women with the TT allele showed a greater difference in their SBP values compared to participants with the AT allele. Of the group of participants homozygous for the TT allele, those with a potassium intake ≥3500 mg per day showed lower differences in their SBP values compared to those with a daily potassium intake <3500 mg.

## 4. Discussion

In this cross-sectional study, we identified an interaction between *FGF5* rs16998073 variants and sodium/potassium intake, the sodium–potassium ratio, and increased risk for hypertension. Both men and women with the minor T alleles (AT or TT allele) for rs16998073 showed significantly increased risks for developing hypertension regardless of sodium/potassium intake or their sodium–potassium ratio when compared to wild-type participants. The protective effect of potassium was seen only in participants with wild-type AA rs16998073 alleles in women with a potassium intake ≥3500 mg per day. The sodium-to-potassium ratio did not show any linear relationship with hypertension, but there was an increased risk for hypertension in patients with mutant rs16998073 at higher sodium–potassium ratios.

Excessive sodium intake is still prevalent in Korea and around the world, although there has been a sustained effort to reduce sodium intake. It is widely known that reduced sodium intake not only lowers blood pressure [24,25] but also reduces cardiovascular morbidity and mortality [26]. Our results were consistent with the fact that excessive sodium intake is known to impact the risk of developing hypertension.

Potassium is the most important dietary factor for lowering blood pressure [7,24], with several studies demonstrating a reduced risk for hypertension-related diseases with increased potassium consumption [27,28]. However, only the *FGF5* rs16998073 wild-type AA allele in women with a potassium intake ≥3500 mg per day demonstrated any protective effect for potassium in hypertension. Therefore, our results do not provide any significant insight into the effects of potassium intake on *FGF5* rs16998073 variants and their risk of hypertension. These inconsistencies can be explained by the fact that the number of participants in this study ingesting ≥3500 mg potassium per day was only one-tenth of the total population. It is possible that this disproportionate representation made it difficult to tease out any interaction between potassium intake and the risk of hypertension.

The sodium–potassium ratio has recently been described as a reliable index for hypertension [8]. A previous study completed in Korea showed that there is a strong association between the sodium–potassium ratio and the prevalence of hypertension in this population [29]. This is in the line with the effect of the urinary sodium–potassium ratio on blood pressure described in a few other studies [29,30]. This is supported by the results of the linear dose–response relationship between the sodium–potassium ratio and systolic blood pressure established in another study [29]. Taken together, these studies support its application as a predictive index for hypertension. However, our results did not confirm a linear relationship between hypertension and the sodium–potassium ratio, but we did demonstrate an increased risk of hypertension for higher sodium–potassium ratios. This suggests that more widespread study is still needed to confirm the utility of this ratio as a predictive index for hypertension.

Blood pressure is primarily controlled by the renin–angiotensin system (RAS), which is affected by the intake of sodium, potassium, and other electrolytes. There are sex-dependent differences in the expression of the RAS components and thus in hypertension. When blood pressure decreases, angiotensinogen is converted to angiotensin I by renin, made in the kidneys, and angiotensin I is converted to angiotensin II by angiotensin converting enzyme. Then angiotensin II interacts with one of its two receptors: angiotensin II type I receptor (AT1R) or angiotensin II type 2 receptor (AT2R). These two receptors perform opposite physiological functions: when angiotensin II combines with AT1R it causes the constriction of the blood vessels, while its combination with AT2R induces vascular expansion [31]. One experimental study showed that male rats have a greater proportion of AT1R and female rats have a greater proportion of AT2R on their surface [31], which suggests that there is fundamental difference in RAS-dependent responses between the two sexes. In our study, a homozygous TT carrier consuming ≥2000 mg of sodium per day had a significantly increased risk for hypertension irrespective of sex, but when the odds ratios for men and women were compared, men were shown to be significantly more at risk for hypertension than women even in this group of participants (AOR = 2.41, 95% CIs = 1.84–3.15, *p*-interaction < 0.0001 vs. AOR = 1.33 95% CIs = 1.09–1.62, *p*-interaction < 0.0001). Additionally, the endocrine system of women is substantially different before and after menopause. So there are studies that show that the female sex hormones may play an important role in preventing hypertension [32]. Other experimental studies have shown that estrogen alters the incidence of hypertension in ovariectomized female rats [33] and offsets the fact that these female animals are more reactive to angiotensin II stimulation than their male counterparts [31]. This is supported by the fact that there is a higher proportion of hypertension in men than in pre-menopausal women but an increased incidence of hypertension in post-menopausal women [34]. However, the mechanism underlying the effects of the sex hormones on blood pressure is not fully understood and requires further evaluation.

The *FGF* gene family, including *FGF5*, has various functions in the human body including tissue development, tissue regeneration, angiogenesis, neoplastic transformation [35], and embryonic development [36]. A recent study showed an abnormal expression of *FGF5* mRNAs and proteins in hypertensive populations, suggesting that *FGF5* may be involved in the development of hypertension [37]. As shown by our results, the genetic effect of *FGF5* rs1698073 might constitute a greater predisposition to hypertension over sodium/potassium intake. In addition, a Bayesian-network-based predictive model for hypertension suggests that *FGF5* rs16998073 could be a deciding factor in the development of hypertension [38]. This model demonstrated an association between hypertension, *FGF5* rs16998073, and urinary albumin [38]. Additionally, salt sensitivity is one of the most important indicators in the development of hypertension and is affected by age, race, and genetic factors [39], with a previous study linking salt sensitivity and *FGF5* in the Korean population [40] with *FGF5* rs16998073 showing the highest likelihood of developing salt sensitivity in an addictive model. These findings are consistent with the results of the present study and suggest that hypertension may be directly affected by salt sensitivity and variations in the *FGF5* rs16998073 locus. However, a number of other studies focused on identifying causative genes for hypertension have identified the genetic expression of *C4orf22* (chromosome 4 open reading frame 22), which is near *FGF5* and other genes, as a causative gene for hypertension rather than *FGF5* itself [11]. One such study showed that injection with an *ANTXR2* (which resides near *C4orf22*) siRNA resulted in an increase in the blood pressure of mice [41], although other studies have since shown that *ANTXR2* knockout mice did not demonstrate any significant difference in their blood pressure [42]. Another study showed that *FGF5* and *ZNF652* might be related to each other and affected each other’s transcriptional activity, thereby altering the prevalence of hypertension in the Chinese Han population [11]. These data suggest that while there may be some link to the *FGF5* rs16998073 locus, further in-depth study is needed to determine the actual causative gene or genes for hypertension.

There are several limitations to our study including the fact that the sodium and potassium intake values were not accurate, because the information collected by the FFQ can be easily underestimated by the subjects and may not reflect the actual amount of ingested sodium. As this was a cross-sectional study, cause–effect relationships between dietary and genetic factors and their impact on increased or decreased risks for developing hypertension could not be evaluated. Additional studies are needed to evaluate the effect of the *FGF5* rs16998073 variants and their relationship with various dietary factors on the development of hypertension. Despite these limitations, we believe that this study is the first to evaluate the interaction between *FGF5* rs16998073, sodium and potassium intake, and the risk of hypertension in the Korean population. In addition, we were able to control against several confounding variables associated with hypertension, helping to confirm several findings from other studies. Lastly, our findings do provide sufficient evidence to suggest that genotyping the *FGF5* gene may be useful in counseling against a genetic predisposition to hypertension, expanding the panel of genetic markers used to evaluate a patient’s likelihood of developing this condition and helping to commence earlier intervention, preventing hypertensive episodes.

## 5. Conclusions

Our results indicate that the minor T allele of *FGF5* rs16998073 is an indicator of increased risk for hypertension regardless of gender, sodium/potassium intake, and sodium–potassium ratios. Both dietary sodium and the sodium–potassium ratio were positively correlated with increased risk for hypertension, but the interaction values for potassium intake could not affirm its relationship with hypertension. The most important finding of our study is the synergistic effect of *FGF5* rs16998073 variants and sodium intake on the risk of hypertension in both men and women. Restriction of sodium intake is recommended for people with an increased risk of hypertension including genetic predispositions. This study suggests that rs16998073 is one such genotype and further longitudinal research is needed to establish a causal relationship between these alleles and hypertension.

## Figures and Tables

**Table 1:** Description of hypertension-associated SNPs identified by GWAS.

No.	SNP	CHR	Minor Allele	MAF		Gene	Hypertension		SBP (*n* = 23,344)		DBP (*n* = 23,344)	
							(Cases (*n* = 9558); Controls (*n* = 8178))					
				Cases	Controls		OR (95%CI)	Add *P*	Beta ± se	Add *P*	Beta ± se	Add *P*
1	rs16998073	4	T	0.50	0.47	*FGF5*	1.29 (1.23–1.35)	8.02 *×* 10^−25^	1.14 ± 0.14	5.46 *×* 10^−17^	0.73 ± 0.09	1.95 *×* 10^−15^
2	rs1458038	4	T	0.36	0.39		1.26 (1.21–1.33)	2.93 *×* 10^−22^	1.05 ± 0.13	1.41 *×* 10^−15^	0.68 ± 0.09	2.77 *×* 10^−14^
3	rs77768175	12	G	0.41	0.37	*HECTD4*	0.77 (0.72–0.82)	5.89 *×* 10^−16^	−0.9 ± 0.18	2.79 *×* 10^−7^	−0.75 ± 0.12	2.92 *×* 10^−10^
4	rs671	12	A	0.38	0.33	*ALDH2*	0.77 (0.72–0.82)	2.08 *×* 10^−15^	−0.89 ± 0.18	3.99 *×* 10^−7^	−0.74 ± 0.12	3.77 *×* 10^−10^
5	rs11066280	12	A	0.23	0.20	*HECTD4*	0.78 (0.74–0.84)	3.15 *×* 10^−14^	−0.83 ± 0.17	1.15 *×* 10^−6^	−0.72 ± 0.12	4.86 *×* 10^−10^
6	rs2074356	12	A	0.30	0.32	*HECTD4*	0.78 (0.73–0.83)	2.86 *×* 10^−13^	−0.89 ± 0.18	1.14 *×* 10^−6^	−0.72 ± 0.12	6.12 *×* 10^−9^
7	rs36034102	4	T	0.32	0.35	*FGF5*	1.22(1.15–1.29)	3.20 *×* 10^−12^	0.87 ± 0.16	3.80 *×* 10^−8^	0.47 ± 0.11	9.21 *×* 10^−6^
8	rs12229654	12	G	0.23	0.26		0.79 (0.74–0.84)	4.25 *×* 10^−25^	−0.9 ± 0.18	1.02 *×* 10^−6^	−0.7 ± 0.12	1.52 *×* 10^−8^
9	rs11066453	12	G	0.24	0.26		0.79 (0.74–0.85)	6.66 *×* 10^−11^	−0.41 ± 0.19	3.41 *×* 10^−2^	−0.42 ± 0.13	1.33 *×* 10^−3^
10	rs2072134	12	A	0.24	0.26	*OAS3*	0.78 (0.73–0.84)	1.278 *×* 10^−10^	−0.49 ± 0.2	1.55 *×* 10^−2^	−0.46 ± 0.14	7.10 *×* 10^−4^
11	rs17249754	12	A	0.24	0.27	*ATP2B1*	0.87 (0.83–0.91)	4.40 *×* 10^−9^	−0.72 ± 0.13	4.69 *×* 10^−8^	−0.39 ± 0.09	1.12 *×* 10^−5^
12	rs12413409	10	A	0.36	0.39	*CNNM2*	0.85 (0.81–0.9)	4.63 *×* 10^−9^	−0.61 ± 0.15	3.73 *×* 10^−5^	−0.33 ± 0.1	7.66 *×* 10^−4^
13	rs16849273	2	G	0.36	0.39		0.87 (0.83–0.91)	4.84 *×* 10^−9^	−0.65 ± 0.13	9.37 *×* 10^−7^	−0.36 ± 0.09	8.19 *×* 10^−5^
14	rs12231049	12	G	0.37	0.39	*MYL2*	0.83 (0.78–0.89)	7.76 *×* 10^−9^	−0.62 ± 0.17	2.60 *×* 10^−4^	−0.46 ± 0.11	6.10 *×* 10^−5^
15	rs732998	10	C	0.16	0.18	*NT5C2*	0.85 (0.81–0.9)	8.48 *×* 10^−9^	−0.59 ± 0.15	5.85 *×* 10^−5^	−0.32 ± 0.1	1.24 *×* 10^−3^
16	rs11191548	10	C	0.16	0.18	*CNNM2*	0.86 (0.81–0.9)	1.04 *×* 10^−8^	−0.59 ± 0.15	6.14 *×* 10^−5^	−0.33 ± 0.1	7.54 *×* 10^−4^
17	rs2681492	12	C	0.13	0.15	*ATP2B1*	0.87 (0.83–0.91)	1.54 *×* 10^−8^	−0.68 ± 0.13	2.48 *×* 10^−7^	−0.38 ± 0.09	2.47 *×* 10^−5^
18	rs3782889	12	G	0.14	0.17	*MYL2*	0.84 (0.79–0.89)	2.03 *×* 10^−8^	−0.58 ± 0.17	6.59 *×* 10^−5^	−0.43 ± 0.11	1.46 *×* 10^−4^
19	rs167479	19	T	0.13	0.16	*RGL3*	0.88 (0.84–0.92)	2.37 *×* 10^−8^	−0.51 ± 0.13	6.57 *×* 10^−5^	−0.35 ± 0.09	5.39 *×* 10^−5^
20	rs112735431	17	A	0.15	0.17	*RNF213, LOC100294362*	1.91 (1.52–2.41)	3.02 *×* 10^−8^	3.4 ± 0.65	1.85 *×* 10^−7^	1.54 ± 0.44	4.89 *×* 10^−4^
21	rs1799998	8	G	0.16	0.18	*CYP11B2*	0.87 (0.83–0.91)	3.16 *×* 10^−8^	−0.22 ± 0.14	1.04 *×* 10^−1^	−0.08 ± 0.09	3.66 *×* 10^−1^
22	rs11191580	10	C	0.11	0.13	*NT5C2*	0.86 (0.82–0.91)	3.39 *×* 10^−8^	−0.57 ± 0.15	1.31 *×* 10^−4^	−0.31 ± 0.1	1.73 *×* 10^−3^
23	rs7136259	12	T	0.10	0.12	*ATP2B1*	0.88 (0.84–0.92)	4.89 *×* 10^−8^	−0.63 ± 0.13	1.65 *×* 10^−6^	−0.36 ± 0.09	4.52 *×* 10^−5^

SNP, single nucleotide polymorphism; CHR, chromosome, MAF, minor allele frequency; OR, odds ratio; CI, confidence interval; SBP, systolic blood pressure; DBP, diastolic blood pressure; se, standard error. Odds ratios were calculated after adjusting for age and gender. Blood pressures used in the linear regression, were adjusted for age and gender. Both logistic and linear regressions were conducted using an additive model.

**Table 2:** General characteristics of the study population stratified by gender.

	Men						Women				
	HTN (*n* = 3575)		No HTN (*n* = 2280)		*p* Value		HTN (*n* = 4355)		No HTN (*n* = 7052)		*p* Value
Age (years)	57.55 ± 7.89		53.66 ± 8.09		<0.0001	Age (years)	57.35 ± 6.91		50.48 ± 7.08		<0.0001
Smoking Status					<0.0001	Smoking Status					<0.0001
Current	796	22.30%	768	33.70%		Current	56	1.30%	165	2.30%	
Past	1688	47.20%	863	37.90%		Past	48	1.00%	73	1.00%	
None	1091	30.50%	649	28.50%		None	4251	97.60%	6814	96.60%	
Physical Activity					<0.0001	Physical Activity					0.916
No	1323	37.00%	962	42.80%		No	2052	47.10%	3322	47.10%	
Yes	2252	63.00%	1318	57.80%		Yes	2303	52.90%	3730	52.90%	
Alcohol Consumption					<0.0001	Alcohol Consumption					<0.0001
Current	2596	72.60%	1541	67.60%		Current	1036	23.80%	2281	32.30%	
Past	304	8.50%	173	7.60%		Past	95	2.20%	106	1.50%	
Never	675	18.90%	566	24.80%		Never	3224	74.00%	4665	66.20%	
BMI (kg/m^2^)	25.22 ± 2.72		23.47 ± 2.54		<0.0001	BMI (kg/m^2^)	24.96 ± 3.04		22.74 ± 2.58		<0.0001
Waist Circumference	87.83 ± 7.33		83.24 ± 7.20		<0.0001	Waist Circumference	81.85 ± 8.05		75.64 ± 7.47		<0.0001
Na Intake (mg/day)	2667.27 ± 1468.11		2624.73 ± 1490.0.4		0.169	Na Intake (mg/day)	2347.85 ± 1328.80		2384.80 ± 1314.68		0.058
K Intake (mg/day)	2244.55 ± 945.03		2248.00 ± 962.94		0.858	K Intake (mg/day)	2168.86 ± 971.31		2262.50 ± 1006.48		<0.0001
Na–K Ratio	1.18 ± 0.39		1.16 ± 0.38		0.042	Na–K Ratio	1.08 ± 0.39		1.06 ± 0.36		0.004
Triglyceride (mg/dL)	160.64 ± 111.76		128.03 ± 88.36		<0.0001	Triglyceride (mg/dL)	134.82 ± 87.20		100.17 ± 62.48		<0.0001
HDL Cholesterol (mg/dL)	48.13 ± 11.42		49.24 ± 11.84		<0.0001	HDL Cholesterol (mg/dL)	52.80 ± 12.42		56.20 ± 12.77		<0.0001
Total Cholesterol (mg/dL)	189.85 ± 34.95		189.61 ± 33.99		0.670	Total Cholesterol (mg/dL)	201.02 ± 36.25		195.54 ± 34.67		<0.0001

All values are expressed as mean ± standard deviation for continuous variables or subject number and percentage of total for categorical variables. HTN, hypertension; HDL, high-density lipoprotein; BMI, body mass index. The *p* values were evaluated using Mann–Whitney U test for the continuous variables and chi-square tests for the categorical variables.

**Table 3:** Interactions between *FGF5* rs16998073 variants and daily sodium/potassium intake and their associated risk for hypertension, stratified on gender.

Men				Women			
	**Na intake**				**Na intake**		
	**<2000 mg**	**≥2000 mg**	***p*-interaction**		**<2000 mg**	**≥2000 mg**	***p*-interaction**
rs16998073			<0.0001	rs16998073			<0.0001
AA (wild type)	1 (ref.)	1.11 (0.92–1.34)		AA (wild type)	1 (ref.)	0.90 (0.78–1.04)	
AT	1.51 (1.24–1.85)	1.62 (1.35–1.95)		AT	1.22 (1.05–1.40)	1.25 (1.09–1.43)	
TT	2.04 (1.48–2.83)	2.41 (1.84–3.15)		TT	1.67 (1.35–2.07)	1.33 (1.09–1.62)	
	**K intake**				**K intake**		
	**<3500 mg**	**≥3500 mg**	***p*-interaction**		**<3500 mg**	**≥3500 mg**	***p*-interaction**
rs16998073			<0.0001	rs16998073			<0.0001
AA (wild type)	1 (ref.)	0.99 (0.75–1.34)		AA (wild type)	1 (ref.)	0.75 (0.58–0.95)	
AT	1.48 (1.30–1.68)	1.48 (1.09–2.01)		AT	1.27 (1.15–1.41)	1.32 (1.05–1.65)	
TT	2.15 (1.74–2.64)	1.85 (0.98–3.48)		TT	1.55 (1.34–1.80)	1.19 (0.77–1.84)	

Adjusted odds ratios (AORs) and 95% confidence intervals were calculated using a multivariable logistic regression model, adjusted for examination site, age, history of smoking or drinking, exercise, and BMI. Participants were classified in six groups based on their sodium/potassium intake and genotype.

**Table 4:** Interaction between *FGF5* rs16998073 variants and sodium–potassium ratios and their impact on hypertension.

Men					Women				
	Na–K Ratio					Na–K ratio			
	Tertile 1	Tertile 2	Tertile 3	*p*-Interaction		Tertile 1	Tertile 2	Tertile 3	*p*-Interaction
rs16998073				<0.0001	rs16998073				<0.0001
AA (wild type)	1 (ref.)	1.17 (0.94–1.46)	1.34 (1.08–1.67)		AA (wild type)	1 (ref.)	1.03 (0.87–1.22)	1.02 (0.56–1.20)	
AT	1.55 (1.25–1.91)	1.86 (1.80–2.31)	1.80 (1.45–2.24)		AT	1.34 (1.14–1.58)	1.24 (1.05–1.46)	1.42 (1.21–1.68)	
TT	2.26 (1.61–3.18)	3.03 (2.14–4.29)	2.12 (1.55–3.12)		TT	1.82 (1.43–2.33)	1.56 (1.22–1.99)	1.40 (1.09–1.79)	

Adjusted odds ratios (AORs) and 95% confidence intervals were calculated using multivariable logistic regression model, adjusted for examination site, age, history of smoking or drinking, exercise, and BMI. Participants were placed into 9 groups based on the tertile value for their sodium–potassium ratio and genotype.
